# Adolescent Pornography Use and Dating Violence among a Sample of Primarily Black and Hispanic, Urban-Residing, Underage Youth

**DOI:** 10.3390/bs6010001

**Published:** 2015-12-23

**Authors:** Emily F. Rothman, Avanti Adhia

**Affiliations:** 1 Department of Community Health Sciences, Boston University School of Public Health, 801 Massachusetts Ave., Floor 4, Boston, MA 02118, USA; 2 Department of Social and Behavioral Sciences, Harvard T. H. Chan School of Public Health, 677 Huntington Avenue, Kresge Building, Boston, MA 02115, USA; aba567@mail.harvard.edu

**Keywords:** pornography, sexually explicit material, adolescent health, dating abuse, partner violence, partner abuse, dating violence

## Abstract

This cross-sectional study was designed to characterize the pornography viewing preferences of a sample of U.S.-based, urban-residing, economically disadvantaged, primarily Black and Hispanic youth (*n* = 72), and to assess whether pornography use was associated with experiences of adolescent dating abuse (ADA) victimization. The sample was recruited from a large, urban, safety net hospital, and participants were 53% female, 59% Black, 19% Hispanic, 14% Other race, 6% White, and 1% Native American. All were 16–17 years old. More than half (51%) had been asked to watch pornography together by a dating or sexual partner, and 44% had been asked to do something sexual that a partner saw in pornography. Adolescent dating abuse (ADA) victimization was associated with more frequent pornography use, viewing pornography in the company of others, being asked to perform a sexual act that a partner first saw in pornography, and watching pornography during or after marijuana use. Approximately 50% of ADA victims and 32% of non-victims reported that they had been asked to do a sexual act that their partner saw in pornography (*p* = 0.15), and 58% did not feel happy to have been asked. Results suggest that weekly pornography use among underage, urban-residing youth may be common, and may be associated with ADA victimization.

## 1. Introduction

Approximately 23% of 10–15 year olds U.S. youth intentionally watch internet pornography at least once per year [1]. Debates about the potential harms of youth exposure to pornography have been raging for decades, but over the past several years, there has been an increasing level of anti-internet pornography activism. Some activist groups now allege that any amount of adolescent pornography use fuels sexual and dating violence and the dissolution of relationships. While the scientific evidence supports the contention that exposure to some forms of sexually explicit material may be harmful to youth (for example, may inspire copycat acts of sexualized violence or degradation, or increase sexual aggression [2,3]), the knowledge base regarding youth exposure to contemporary internet-based pornography and sequelae remains too limited. A handful of studies published since the advent of widespread internet availability have found associations between youth exposure to pornography and subsequent sexual aggression, permissive sexual norms and gender role attitudes, earlier sexual behavior, lower levels of sexual satisfaction, higher preferences for certain body types, negative attitudes towards monogamy, participation in group sex, and higher numbers of sexual partners [1,2,3,4,5,6,7,8,9,10]. Few of these used research designs that permit causal inferences (for exceptions see [1,2]), and at least three recent studies have found null or inconclusive evidence of a relationship between exposure to pornography and adolescent sexual risk taking or sexual compulsivity [11,12,13]. 

One problem with the debate about the potential harms of pornography is that it presupposes all pornography is alike. In actuality, pornography is now available in dozens of genres and is consumed in a wide variety of settings (e.g., alone at home, at school with friends), for a number of different purposes (e.g., for masturbation, to relieve boredom, out of curiosity), and under various conditions (e.g., sober *vs*. intoxicated, coerced *vs*. consensual). Consumers of pornography—even youth consumers—differ from one another in terms of culture, preexisting knowledge, attitudes about sex and sexually explicit material, parental monitoring and/or guidance pertaining to pornography, sexual orientation, and personality traits, and these may moderate the impact of pornography on outcomes of interest [14]. Research on the impact of pornography on youth has tended to overlook the diversity of pornographies and oversimplify, potentially leading to incorrect or under-nuanced conclusions about whether, how, and why some forms of sexually explicit material may be impacting the psychology and behavior of some youth [15]. 

Evidence on pornography and its health impact will be substantially improved when the types of sexually explicit material are more clearly defined in research studies, and when the knowledge base includes information about youth that have been underrepresented. The majority of pornography research has been conducted using samples of college-attending, or primarily White, European youth [4,5,16,17]. Research describing the pornography preferences and use patterns of economically disadvantaged, urban, youth of color is particularly lacking. This is an important omission from the field, because “youth” are not monolithic—in fact, adolescent sexual behavior varies across subgroups of youth by age, gender, culture, sexual orientation, and religiosity [2,18]. Therefore, the results of pornography studies that have failed to include poor youth or youth of color cannot necessarily be generalized to all youth. Increased research attention to the potential impact of pornography use on youth of color should be prioritized given the national goal of reducing race- and ethnicity-based health disparities. Economically disadvantaged youth, and Black and Hispanic youth, are at increased risk for experiencing unintended pregnancy, acquiring sexually transmitted infections (STIs) including HIV, and relationship violence as compared to wealthy or White counterparts [19,20,21,22]. Sexual content in media can affect Black and White youth differently [23], and the results of multiple studies suggest that Black youth and adults are more likely to be exposed to pornography than their White counterparts [2,24]. Race, ethnicity, class, and culture may have salience in terms of understanding the possible chain of events that could lead from sexually explicit media exposure, to youth’s interpretation or engagement with that media, to exacerbated risk for undesirable health outcomes. Thus, understanding the pornography use preferences and patterns of economically disadvantaged youth of color could inform sexual and reproductive health promotion, and possibly sexual relationship safety, by informing the development of culturally-relevant educational and intervention programs. 

Sexual relationship safety, the converse of adolescent dating abuse (ADA), is now recognized as one of the most pressing public health problems in the nation. Approximately 21% of girls and 10% of boys who attend high school in the U.S. experience either physical or sexual ADA each year [25]. Sequelae of ADA can be severe and may include depression, anxiety, substance abuse, antisocial behavior, suicidal thoughts, injury, and death [26,27,28]. ADA victimization is also predictive of subsequent repeat victimization [26,29]. For these reasons, ADA has been declared a public health priority by the U.S. Centers for Disease Control and Prevention, and the Obama administration [30,31]. The results of prior qualitative research suggest that ADA and the viewing of sexually explicit images could be linked reciprocally, although that association has yet to be demonstrated using quantitative data [6]. Therefore, the present study was designed to characterize the pornography viewing preferences of a sample of U.S-based, urban-residing, economically disadvantaged youth, and to explore whether ADA victimization status was associated with pornography use choices and experiences. 

## 2. Methods

### 2.1. Sample and Setting

A convenience sample of youth was recruited from a large safety net hospital in Boston, Massachusetts. Safety net hospitals are health care entities that primarily serve low income and uninsured patients. Patients were eligible if they were between 16–17 years old (*i.e.*, younger than the legal age to view pornography online), medically stable, English-speaking, and reported having seen pornography at least twice in the past year either intentionally or unintentionally. Parental consent was obtained from youth whose guardians accompanied them to the hospital; youth who were 16 years old or older and consented to their own medical treatment were also able to assent to their own participation in this research. Participation was anonymous. Of 198 patients screened, 43% were eligible and of those eligible, 93% enrolled. All procedures were approved by the hospital Institutional Review Board (IRB).

Participant recruitment took place January 2013–January 2014. A research assistant (RA) monitored a computer system that revealed when patients in the appropriate age range were waiting for treatment. When they were brought to a treatment room, the RA approached those patients and explained that they might have an opportunity to participate in a research study if they were interested, and invited them to complete eligibility surveys. Those who were eligible were subsequently provided with details about participation and asked for assent, then completed the self-administered, paper-based questionnaire. If study participants were accompanied by a parent, friend, or other individual, that person was asked to wait outside of the private treatment room where data were collected. If participants did not understand the precise meaning of a question, the RA was standing by in order to clarify. Study participants (*n* = 79) were 53% female, 59% Black, 19% Hispanic, 14% Other race, 6% White, and 1% Native American. Eighty percent reported that they were heterosexual. Because seven participants were missing data on the dating abuse victimization variable, the analytic sample was 72 participants.

### 2.2. Measures

The survey comprised 59 questions and took participants roughly 30 min to complete. Demographic information on participants’ gender, age, race/ethnicity, sexual orientation, education level, and relationship status was collected via standard questions (*i.e.*, those asked on the national or state versions of the Youth Risk Behavior Survey). 

#### 2.2.1. Dating Abuse Victimization 

Past year ADA victimization was assessed via an 11-item version of the Severity of Violence Against Women Scales (SVAWS) [32]. Sample items included the question stem “In the past year, how many times has a partner…” and “hit or kicked a wall, door, or furniture”, or “demanded sex whether you wanted it or not.” The scale reliability coefficient in this sample was α = 0.81. 

#### 2.2.2. Pornography Use Frequency

Pornography use frequency was assessed using three questions. An example item was: “about how many times have you viewed, seen, or used pornography in the past week? (Count internet, movies, magazines, books).” The word “pornography” was defined as “X-rated” on the eligibility form; cognitive interviewing during a prior qualitative study using a sample recruited from the same setting revealed that the study population clearly understood the term “pornography” to mean sexually explicit material. 

#### 2.2.3. Pornography Website Preference 

Respondents were asked: “Thinking about the times you have seen internet pornography in the past year, which websites did you visit one or more times? (Check all that apply).” Response options included the most popular free pornography sites from 2012, which included: YouPorn, Pornhub, Pornsite, Redtube, Zootube, and ‘other’ with a line to fill in.

#### 2.2.4. Pornography Category Preference 

Respondents were asked: “Some pornography websites have different categories or types of pornography you can choose. Which of the following types do you choose MOST often? (Please check the top 3–4 that you enjoy, or try to view, the most often).” There were 62 different categories presented, in addition to “none of the above” and an option to fill in an “other” category. The 62 categories were compiled from the home pages of the YouPorn, Pornhub, and Pornsite websites. Examples of the 62 categories were: amateur, anal, creampie, ebony, fetish, group sex, hentai, lesbian, MILF, teen, threesome, vintage, and webcam. 

#### 2.2.5. Partner Behaviors and Pornography 

Respondents were asked: “have you ever been in a situation where your boyfriend/girlfriend (or any sex partner) asked you to do something that he or she first saw in pornography?” Response options were “yes”, “no”, and “don’t know”. Participants who responded affirmatively were asked, “How did you feel about what your partner asked you to do?” Response options included “Happy: I liked being asked to do that”; “Sad: I did not like being asked to do that”; and “I don’t know”. Respondents were also asked “Has a dating or sex partner of yours ever asked you to watch pornography together?” with response options of “yes”, “no” and “don’t know”. 

#### 2.2.6. Sources of Information about Sex 

Respondents were asked to identify their primary source of information about sex by ranking a set of eight options. They were asked: “Thinking about your life, which of these things have taught you the most about sex? Put them in order where you give a 1 to the thing that taught you the most, and an 8 to the one that taught you the least.” Response options included “my parents or guardians”, “a doctor”, “school or teachers at school”, “my pastor or religious leader”, “my brother, sister, cousin or friends (kids my age)”, “pornography”, “TV or movies (non-pornography)”, and “other”. A dummy variable representing the primary source of information was created reflecting the response option to which each respondent assigned a value of one. 

### 2.3. Analytic Procedures

We used STATA version 13.1 for statistical analysis. We tabulated descriptive information on ADA victimization for the full sample. Responses to survey questions were determined to be missing at random, so analyses were conducted on non-missing data. Pearson’s Chi-square statistic was used to assess the statistical significance of differences in pornography-related behaviors by ADA victimization status for categorical data, and t-tests were used for continuous data. Due to the relatively small sample size and exploratory nature of the study, a cut-point of *p* < 0.10 was used to avoid Type II error [33,34,35]. In one instance, we considered the difference between two proportions of possible clinical relevance when the p-value was equal to 0.15. One-sided Fisher’s exact test statistic was used when cell counts were ≤ 5. Because there were no differences in ADA victimization by gender or race, analyses did not control for these factors [36]. Because Bonferroni adjustments to control for Type I error “are, at best, unnecessary and, at worst, deleterious to sound statistical inference,” [37] we opted not to “correct” for having carried out multiple tests. The debate about correcting for multiple tests is beyond the scope of this paper [38].

## 3. Results

### 3.1. Pornography Use

On average, participants reported watching pornography approximately twice per week, and 51% reported watching pornography weekly or more often (Table 1). They were as likely to report watching pornography with other people as alone (Table 1). The most preferred website was Pornhub (63%), followed by YouPorn (18%), and Pornsite (11%). Participants reported that they were most likely to select to view lesbian/bisexual or “big butt/big tits” pornography (44% and 43%, respectively), followed by Ebony or Latina pornography (39%), blowjobs (21%), threesomes (16%), and teen sex (13%) (Table 1). More than half the sample (56%) reported ever trying a sexual act because they first saw it in pornography, and 54% reported ever watching pornography in order to learn how to do something sexual. More than half (51%) had ever been asked to watch pornography together by a dating or sexual partner, and 44% had ever been asked to do something sexual that a partner first saw in pornography (Table 1). Approximately 61% of those who had been asked to watch pornography together by a dating or sexual partner were also asked to do something sexual that they believed a partner first saw in pornography. Approximately 43% had ever watched pornography during or after marijuana use, and 17% had watched pornography during or after alcohol use. Approximately 10% reported that they had ever watched pornography while inside a public high school, and 6% reported that they had ever asked a sibling or other person younger than 18 years old to watch pornography (Table 1). 

**Table 1:** Prevalence of pornography-related behaviors, by dating violence victimization status (*n* = 72).

Behaviors	Total sample % (n)	No dating abuse in past year % (n)	Dating abuse in past year % (n)	χ^2^ or t-test *p*-value
Total	100% (72)	100% (22)	100% (50)	
Frequency of pornography viewing [continuous variable]				
No. of times per week (mean, SD)	1.99 (3.34)	1.14	2.36	0.06 *
No. of times per month (mean, SD)	6.90 (12.45)	3.77	8.28	0.05 **
No. of times per year (mean, SD)	29.83 (54.45)	13.95	37.10	0.02 **
Weekly use [categorical responses]				
0 times per week	49% (35)	59% (13)	44% (22)	0.24
1–9 times per week	46% (33)	41% (9)	48% (24)	0.58
10–20 times per week	6% (4)	0% (0)	8% (4)	0.17
Monthly use [categorical responses]				
0 times per month	21% (15)	23% (5)	20% (10)	0.79
1–9 times per month	57% (41)	64% (14)	54% (27)	0.45
10–19 times per month	13% (9)	14% (3)	12% (6)	0.85
20 or more times per month	10% (7)	0% (0)	14% (7)	0.07 *
Yearly use [categorical responses]				
1–9 times per year	38% (27)	41% (9)	36% (18)	0.69
10–99 times per year	39% (28)	50% (11)	34% (17)	0.20
100 or more times per year	10% (7)	0% (0)	14% (7)	0.07 *
With whom pornography is most often watched				
Alone	51% (35)	62% (13)	46% (22)	0.22
Intimate partner	29% (20)	24% (5)	31% (15)	0.37
Friend(s) or family members	20% (14)	14% (3)	23% (11)	0.32
Preferred internet pornography website				
Pornhub	63% (45)	68% (15)	60% (30)	0.51
YouPorn	18% (13)	18% (4)	18% (9)	0.61
Pornsite	11% (8)	0% (0)	16% (8)	0.05**
Redtube	8% (6)	5% (1)	10% (5)	0.40
Zootube	3% (2)	0% (0)	4% (2)	0.48
Types of pornography chosen most often ^†^				
Lesbian/Bisexual	44% (31)	36% (8)	48% (23)	0.37
Big butt/Big tits	43% (30)	36% (8)	46% (22)	0.46
Ebony/Latina	39% (27)	41% (9)	38% (18)	0.79
Blowjob	21% (15)	18% (4)	23% (11)	0.46
Threesome	16% (11)	5% (1)	21% (10)	0.08*
Group sex	11% (8)	14% (3)	10% (5)	0.49
Teen	13% (9)	18% (4)	10% (5)	0.29
Pornography-related experiences				
Tried something in real life because saw it in porn	56% (40)	45% (10)	60% (30)	0.25
Watched porn in order to learn how to do something sexual	54% (39)	45% (10)	58% (29)	0.33
Asked to watch pornography together by a partner	51% (37)	41% (9)	56% (28)	0.24
Asked to do something partner first saw in pornography	44% (32)	32% (7)	50% (25)	0.15
Felt happy to be asked to do something partner saw in porn^†^	42% (14)	43% (3)	42% (11)	0.85
Watched pornography during or after marijuana use	43% (31)	23% (5)	52% (26)	0.02 **
Watched pornography during or after alcohol use	17% (12)	9% (2)	20% (10)	0.22
Ever been in a pornography video (professional or amateur)	11% (8)	5% (1)	14% (7)	0.23
Watched pornography at a public high school	10% (7)	5% (1)	12% (6)	0.31
Ever asked a sibling or other person < 18 years old to watch porn	6% (4)	0% (0)	8% (4)	0.22
Primary source of education about sex				
Pornography	30% (21)	33% (7)	29% (14)	0.69
Parents/guardians	21% (15)	29% (6)	18% (9)	0.34
Siblings or peers	16% (11)	14% (3)	16% (8)	0.57
School or teachers	11% (8)	14% (3)	10% (5)	0.45
Non-sexually explicit TV or movies	10% (7)	0% (0)	14% (7)	0.07 *
Doctor	9% (6)	10% (2)	8% (4)	0.59
Other	3% (2)	0% (0)	4% (2)	0.49

Notes: * *p* ≤ 0.10, ** *p* ≤ 0.05; ^†^ Only asked of those who had ever been asked to do something partner saw in pornography.

### 3.2. Dating Abuse Victimization

Of this sample of 72 youth, 69% had experienced at least one instance of physical or sexual dating abuse in the past year (*n* = 50). Females and males were equally likely to report ADA victimization (70% and 69%, respectively), and there were no substantial differences by race, sexual orientation, level of education, or partnership-status. 

### 3.3. Dating Abuse Victimization and Pornography Use

ADA victims watched pornography more frequently than non-victimized counterparts. ADA victims reported viewing pornography approximately twice as often per week (2.4 *vs*. 1.1 times, *p* = 0.06), twice as often per month (8.3 *vs*. 3.8 times, *p* = 0.05), and approximately 2.6 times more frequently per year (37.1 *vs*. 14.0 times, *p* < 0.05) than non-victimized participants (Table 1). ADA victims were also more likely to report viewing pornography with other people (rather than alone) (54% *vs*. 38%, *p* < 0.01), to report that they had ever been asked to do a sexual act that they believed their partner first saw in pornography (50% *vs*. 32%, *p* = 0.15), and that they had ever watched pornography during or after marijuana use (52% *vs*. 23%, *p* < 0.05). Approximately 42% of those asked to do something that they believed their partner first saw in pornography reported feeling “happy” to have been asked. Those who reported ADA victimization were also more likely than those with no ADA victimization to report that their preferred pornography website was Pornsite (16% *vs*. 0%, *p* = 0.05), and that the type of pornography that they most often chose to watch involved threesomes (21% *vs*. 5%, *p* < 0.10). All of the participants who had ever asked a sibling or other person younger than 18 years old to watch pornography were ADA victims (*n* = 4), and all but one of the participants who had ever watched pornography at a public high school were ADA victims (*n* = 6). The primary source of information about sex for both ADA victims and non-victims was pornography (30%). ADA victims and non-victims were almost equally likely to rank pornography as their primary source of information about sex (Table 1). However, ADA victims were more likely than non-victims to report that non-sexually explicit TV or movies were a primary source of information about sex (14% *vs*. 0%, *p* < 0.10), and less likely to report that parents or guardians were primary sources of information (18% *vs*. 29%, NS). 

## 4. Discussion

This was an exploratory study designed as a preliminary effort to describe pornography use among a sample of youth at high risk for experiencing health disparities who have been underrepresented in pornography-related research—that is, urban-residing, economically disadvantaged, underage youth, almost all of whom (94%) were of color. It is an important contribution to the literature because little is known about the pornography use preferences of urban-residing, economically disadvantaged, underage youth, and this study establishes a “starting point” for undertaking additional empirical research by generating estimates that can be used for power calculations and descriptive statistics for future empirical work.

Participants reported, on average, watching pornography as frequently as twice per week. This rate is almost identical to the rate reported for one primarily White, slightly older, and college-attending sample; Carroll et al. found that in a sample of 813 university students (79% White), approximately half (50%) reported viewing pornography weekly or more often [17]. However, compared to one other study of urban, primarily Black and Hispanic youth, those in our sample reported more frequent pornography use. Braun-Courville and Rojas found that 70% of a primarily female, Black and Hispanic, New York City health clinic sample reported less than monthly exposure to sexually explicit internet material in the preceding three months [39]. Differences in when the research was conducted (2009 *vs*. 2014), proportion of females in study samples, and the interpretability of survey questions may account for the variation.

The findings that more than half the sample (56%) reported that they had ever tried a sexual act because they first saw it in pornography, and 54% reported ever watching pornography in order to learn how to do something sexual are consistent with prior studies that have found that some youth use sexually explicit material to learn about sexual organs and functions, the mechanisms of sex, and to further sexual identity development [6,40,41,42,43]. In addition, the results of this study suggest that being asked to watch pornography together with a dating or sexual partner may be common for some underage youth, and lends support to the contention that the majority of youth who are asked to watch pornography with a partner are subsequently asked to reenact something depicted. This information could inform pornography literacy curricula developed for urban youth of color [44]: the results of this particular study suggest that if your partner asks you to view pornography with him or her, there is a high likelihood that you will also be asked to do something that he or she has seen in pornography. Youth could be taught to be prepared for such requests and to decide where their boundaries are related to particular sex acts in advance. Because less than half of those asked to reenact something from pornography reported feeling happy about being asked (42%), being prepared to be asked may help youth formulate responses that keep them safe [6]. Furthermore, all youth in this sample were younger than the minimum age for viewing internet pornography (*i.e.*, 18 years old), so being induced to watch by a dating partner carried at least some legal risk for both parties. Again, pornography literacy curricula could include information about possible legal risks of inducing a minor to watch sexually explicit internet material. Given that 30% of this sample reported that pornography was their primary source of information about sex, calls to expand the reach of sex education and that pornography literacy be developed as components of it are supported [44,45]. 

To our knowledge, this is the first quantitative study to suggest that ADA victimization may be associated with more frequent pornography use, viewing pornography in the company of others, being asked to perform a sexual act that a partner first saw in pornography, and watching pornography during or after marijuana use. The relationship between ADA and pornography use merits further study. It is possible that ADA victims were coerced or forced to watch more pornography or perform pornography-inspired acts by ADA perpetrators, but it is also possible that their pornography use (whether self-directed or directed by an individual other than a dating or sexual partner) predisposed them to ADA victimization, or that the association reflects a third, unmeasured factor. Determining the strength and nature of the association through larger-scale, nationally-representative, and longitudinal research would benefit the field. Nevertheless, at a minimum these findings suggest that clinicians, educators, parents, and other youth-serving professionals need guidance about how to talk factually with youth about present-day pornography, its potential impacts, and what they can do if they are being pressured to watch or perform pornographic acts. 

The results of this study also suggest that additional investigation into the relationships between underage alcohol use, marijuana use, and pornography viewing are needed. While nearly half (43%) of youth in this sample reported ever viewing pornography during or after using marijuana, only 17% had watched pornography during or after alcohol use. It is unclear why marijuana use would have a stronger relationship to pornography viewing than alcohol, though several possibilities exist. A smaller proportion of Black youth consume alcohol than do White youth, so the relationship may simply reflect substance use patterns of this sample [46]. On the other hand, when youth consume alcohol they may be more likely to do so in party or social group settings that are less conducive to pornography viewing. It is also possible that marijuana use increases youths’ interest in viewing pornography more often than does alcohol consumption.

It is worrisome that 10% of youth in this sample reported viewing pornography in public high schools. As of June 2015, 24 states in the U.S. have laws that mandate public schools and libraries to use internet filtering to prevent youth exposure to sexually explicit material, including the state in which this study was conducted [47]. However, some state laws pertain only to computer terminals provided by schools, and thus do not apply to internet access from students’ personal electronic devices—even though the capability to block certain domain names via wireless access in a given building exists. When youth view pornography in school, it can lead to nonconsensual exposure to the sexually explicit material by other students or, as the results of one recent qualitative study suggest, instigate sexual harassment [6]. It is also of concern that 6% of youth in this sample had ever asked a sibling or other person younger than 18 years old to watch pornography. Inducing underage youth to watch sexually explicit material is not only potentially harmful to the victim, but could create legal or other problems for the perpetrator.

### Limitations

The sample size was modest (*n* = 72), and not representative of all youth in the U.S; the study may have been underpowered to detect some associations between ADA victimization and pornography use. Replication research with a nationally-representative sample would further the field. Second, data were self-reported. It is possible that youth either underreported or overreported their pornography use or ADA victimization experiences. Third, questions about pornography use have not been subjected to psychometric testing. Methodologic research that yields valid, reliable assessment instruments for pornography studies would benefit the field.

## 5. Conclusions 

ADA is a critical public health problem and understanding how to intervene with potential perpetrators and victims effectively is a priority. Based on the preliminary evidence presented here, it makes sense to continue to investigate whether underage (< 18 years old) viewing of sexually explicit media could elevate risk for ADA, if ADA could elevate risk for sexually explicit media viewing, if both are related to some (or several) other factor(s), or if all of those possible explanations are true for different subsets of youth. An important takeaway is that scholarship about pornography and violence should take into account the specific subgenre of sexually explicit material that subjects have viewed or prefer to view, should assess the frequency of exposure, and should consider the possibility that personality traits and other contextual factors (*i.e.*, religiosity, rural vs. urban setting, parental monitoring of internet use) could result in differences in the apparent impact of sexually explicit media use on subsets of youth. The developmental age, race, ethnicity, socioeconomic status, and culture of the youth participants in pornography and aggression research should be thoughtfully addressed with the ultimate goal of creating effective, evidence-based violence prevention and pornography literacy interventions. 
